# *RAD51B* in Familial Breast Cancer

**DOI:** 10.1371/journal.pone.0153788

**Published:** 2016-05-05

**Authors:** Liisa M. Pelttari, Sofia Khan, Mikko Vuorela, Johanna I. Kiiski, Sara Vilske, Viivi Nevanlinna, Salla Ranta, Johanna Schleutker, Robert Winqvist, Anne Kallioniemi, Thilo Dörk, Natalia V. Bogdanova, Jonine Figueroa, Paul D. P. Pharoah, Marjanka K. Schmidt, Alison M. Dunning, Montserrat García-Closas, Manjeet K. Bolla, Joe Dennis, Kyriaki Michailidou, Qin Wang, John L. Hopper, Melissa C. Southey, Efraim H. Rosenberg, Peter A. Fasching, Matthias W. Beckmann, Julian Peto, Isabel dos-Santos-Silva, Elinor J. Sawyer, Ian Tomlinson, Barbara Burwinkel, Harald Surowy, Pascal Guénel, Thérèse Truong, Stig E. Bojesen, Børge G. Nordestgaard, Javier Benitez, Anna González-Neira, Susan L. Neuhausen, Hoda Anton-Culver, Hermann Brenner, Volker Arndt, Alfons Meindl, Rita K. Schmutzler, Hiltrud Brauch, Thomas Brüning, Annika Lindblom, Sara Margolin, Arto Mannermaa, Jaana M. Hartikainen, Georgia Chenevix-Trench, kConFab/AOCS Investigators, Laurien Van Dyck, Hilde Janssen, Jenny Chang-Claude, Anja Rudolph, Paolo Radice, Paolo Peterlongo, Emily Hallberg, Janet E. Olson, Graham G. Giles, Roger L. Milne, Christopher A. Haiman, Fredrick Schumacher, Jacques Simard, Martine Dumont, Vessela Kristensen, Anne-Lise Borresen-Dale, Wei Zheng, Alicia Beeghly-Fadiel, Mervi Grip, Irene L. Andrulis, Gord Glendon, Peter Devilee, Caroline Seynaeve, Maartje J. Hooning, Margriet Collée, Angela Cox, Simon S. Cross, Mitul Shah, Robert N. Luben, Ute Hamann, Diana Torres, Anna Jakubowska, Jan Lubinski, Fergus J. Couch, Drakoulis Yannoukakos, Nick Orr, Anthony Swerdlow, Hatef Darabi, Jingmei Li, Kamila Czene, Per Hall, Douglas F. Easton, Johanna Mattson, Carl Blomqvist, Kristiina Aittomäki, Heli Nevanlinna

**Affiliations:** 1 Department of Obstetrics and Gynecology, University of Helsinki and Helsinki University Hospital, Helsinki, Finland; 2 Laboratory of Cancer Genetics and Tumor Biology, Cancer Research and Translational Medicine, Biocenter Oulu, University of Oulu, Oulu, Finland; 3 BioMediTech, University of Tampere, Tampere, Finland; 4 Department of Medical Biochemistry and Genetics, University of Turku, Turku, Finland; 5 Tyks Microbiology and Genetics, Department of Medical Genetics, Turku University Hospital, Turku, Finland; 6 Laboratory of Cancer Genetics and Tumor Biology, Northern Finland Laboratory Centre NordLab, Oulu, Finland; 7 Fimlab Laboratories, Tampere, Finland; 8 Gynaecology Research Unit, Hannover Medical School, Hannover, Germany; 9 Radiation Oncology Research Unit, Hannover Medical School, Hannover, Germany; 10 Division of Cancer Epidemiology and Genetics, National Cancer Institute, Rockville, Maryland, United States of America; 11 Centre for Cancer Genetic Epidemiology, Department of Oncology, University of Cambridge, Cambridge, United Kingdom; 12 Centre for Cancer Genetic Epidemiology, Department of Public Health and Primary Care, University of Cambridge, Cambridge, United Kingdom; 13 Netherlands Cancer Institute, Antoni van Leeuwenhoek hospital, Amsterdam, The Netherlands; 14 Division of Genetics and Epidemiology, The Institute of Cancer Research, London, United Kingdom; 15 Centre for Epidemiology and Biostatistics, Melbourne School of Population and Global health, The University of Melbourne, Melbourne, Australia; 16 Department of Pathology, The University of Melbourne, Melbourne, Australia; 17 Department of Gynaecology and Obstetrics, University Hospital Erlangen, Friedrich-Alexander University Erlangen-Nuremberg, Comprehensive Cancer Center Erlangen-EMN, Erlangen, Germany; 18 David Geffen School of Medicine, Department of Medicine, Division of Hematology and Oncology, University of California Los Angeles, Los Angeles, California, United States of America; 19 Department of Non-Communicable Disease Epidemiology, London School of Hygiene and Tropical Medicine, London, United Kingdom; 20 Research Oncology, Guy’s Hospital, King's College London, London, United Kingdom; 21 Wellcome Trust Centre for Human Genetics and Oxford NIHR Biomedical Research Centre, University of Oxford, Oxford, United Kingdom; 22 Department of Obstetrics and Gynecology, University of Heidelberg, Heidelberg, Germany; 23 Molecular Epidemiology Group, German Cancer Research Center (DKFZ), Heidelberg, Germany; 24 Environmental Epidemiology of Cancer, Center for Research in Epidemiology and Population Health, INSERM, Villejuif, France; 25 University Paris-Sud, Villejuif, France; 26 Copenhagen General Population Study, Herlev Hospital, Copenhagen University Hospital, Herlev, Denmark; 27 Department of Clinical Biochemistry, Herlev Hospital, Copenhagen University Hospital, Herlev, Denmark; 28 Faculty of Health and Medical Sciences, University of Copenhagen, Copenhagen, Denmark; 29 Human Cancer Genetics Program, Spanish National Cancer Research Centre, Madrid, Spain; 30 Centro de Investigación en Red de Enfermedades Raras, Valencia, Spain; 31 Beckman Research Institute of City of Hope, Duarte, California, United States of America; 32 Department of Epidemiology, University of California Irvine, Irvine, California, United States of America; 33 Division of Clinical Epidemiology and Aging Research, German Cancer Research Center (DKFZ), Heidelberg, Germany; 34 German Cancer Consortium (DKTK), German Cancer Research Center (DKFZ), Heidelberg, Germany; 35 Division of Preventive Oncology, German Cancer Research Center (DKFZ) and National Center for Tumor Diseases (NCT), Heidelberg, Germany; 36 Division of Gynaecology and Obstetrics, Technische Universität München, Munich, Germany; 37 Center for Hereditary Breast and Ovarian Cancer, University Hospital of Cologne, Cologne, Germany; 38 Center for Integrated Oncology (CIO), University Hospital of Cologne, Cologne, Germany; 39 Center for Molecular Medicine Cologne (CMMC), University of Cologne, Cologne, Germany; 40 Dr. Margarete Fischer-Bosch-Institute of Clinical Pharmacology, Stuttgart, Germany; 41 University of Tübingen, Tübingen, Germany; 42 Institute for Prevention and Occupational Medicine of the German Social Accident Insurance, Institute of the Ruhr University Bochum, Bochum, Germany; 43 Department of Molecular Medicine and Surgery, Karolinska Institutet, Stockholm, Sweden; 44 Department of Oncology—Pathology, Karolinska Institutet, Stockholm, Sweden; 45 Cancer Center, Kuopio University Hospital, Kuopio, Finland; 46 Institute of Clinical Medicine, Pathology and Forensic Medicine, University of Eastern Finland, Kuopio, Finland; 47 Imaging Center, Department of Clinical Pathology, Kuopio University Hospital, Kuopio, Finland; 48 Department of Genetics, QIMR Berghofer Medical Research Institute, Brisbane, Australia; 49 Peter MacCallum Cancer Center, The University of Melbourne, Melbourne, Australia; 50 Vesalius Research Center, VIB, Leuven, Belgium; 51 Laboratory for Translational Genetics, Department of Oncology, University of Leuven, Leuven, Belgium; 52 Leuven Multidisciplinary Breast Center, Leuven Cancer Institute, University Hospitals Leuven, Leuven, Belgium; 53 Division of Cancer Epidemiology, German Cancer Research Center (DKFZ), Heidelberg, Germany; 54 University Cancer Center Hamburg (UCCH), University Medical Center Hamburg-Eppendorf, Hamburg, Germany; 55 Unit of Molecular Bases of Genetic Risk and Genetic Testing, Department of Preventive and Predictive Medicine, Fondazione IRCCS (Istituto Di Ricovero e Cura a Carattere Scientifico) Istituto Nazionale dei Tumori (INT), Milan, Italy; 56 IFOM, Fondazione Istituto FIRC (Italian Foundation of Cancer Research) di Oncologia Molecolare, Milan, Italy; 57 Department of Health Sciences Research, Mayo Clinic, Rochester, Minnesota, United States of America; 58 Cancer Epidemiology Centre, Cancer Council Victoria, Melbourne, Australia; 59 Department of Preventive Medicine, Keck School of Medicine, University of Southern California, Los Angeles, California, United States of America; 60 Genomics Center, Centre Hospitalier Universitaire de Québec Research Center, Laval University, Québec City, Canada; 61 Department of Genetics, Institute for Cancer Research, Oslo University Hospital Radiumhospitalet, Oslo, Norway; 62 K.G. Jebsen Center for Breast Cancer Research, Institute of Clinical Medicine, Faculty of Medicine, University of Oslo, Oslo, Norway; 63 Department of Clinical Molecular Biology, Oslo University Hospital, University of Oslo, Oslo, Norway; 64 Division of Epidemiology, Department of Medicine, Vanderbilt-Ingram Cancer Center, Vanderbilt University School of Medicine, Nashville, Tennessee, United States of America; 65 Department of Surgery, Oulu University Hospital, University of Oulu, Oulu, Finland; 66 Lunenfeld-Tanenbaum Research Institute of Mount Sinai Hospital, Toronto, Canada; 67 Department of Molecular Genetics, University of Toronto, Toronto, Canada; 68 Department of Pathology, Leiden University Medical Center, Leiden, The Netherlands; 69 Department of Human Genetics, Leiden University Medical Center, Leiden, The Netherlands; 70 Department of Medical Oncology, Family Cancer Clinic, Erasmus MC Cancer Institute, Rotterdam, The Netherlands; 71 Department of Clinical Genetics, Erasmus University Medical Center, Rotterdam, The Netherlands; 72 Sheffield Cancer Research, Department of Oncology, University of Sheffield, Sheffield, United Kingdom; 73 Academic Unit of Pathology, Department of Neuroscience, University of Sheffield, Sheffield, United Kingdom; 74 Clinical Gerontology, Department of Public Health and Primary Care, University of Cambridge, Cambridge, United Kingdom; 75 Molecular Genetics of Breast Cancer, German Cancer Research Center (DKFZ), Heidelberg, Germany; 76 Institute of Human Genetics, Pontificia Universidad Javeriana, Bogota, Colombia; 77 Department of Genetics and Pathology, Pomeranian Medical University, Szczecin, Poland; 78 Department of Laboratory Medicine and Pathology, Mayo Clinic, Rochester, Minnesota, United States of America; 79 Molecular Diagnostics Laboratory, IRRP, National Centre for Scientific Research "Demokritos", Athens, Greece; 80 Breakthrough Breast Cancer Research Centre, The Institute of Cancer Research, London, United Kingdom; 81 Division of Breast Cancer Research, The Institute of Cancer Research, London, United Kingdom; 82 Department of Medical Epidemiology and Biostatistics, Karolinska Institutet, Stockholm, Sweden; 83 Department of Oncology, University of Helsinki and Helsinki University Hospital, Helsinki, Finland; 84 Department of Clinical Genetics, University of Helsinki and Helsinki University Hospital, Helsinki, Finland; Odense University Hospital, DENMARK

## Abstract

Common variation on 14q24.1, close to *RAD51B*, has been associated with breast cancer: rs999737 and rs2588809 with the risk of female breast cancer and rs1314913 with the risk of male breast cancer. The aim of this study was to investigate the role of *RAD51B* variants in breast cancer predisposition, particularly in the context of familial breast cancer in Finland. We sequenced the coding region of *RAD51B* in 168 Finnish breast cancer patients from the Helsinki region for identification of possible recurrent founder mutations. In addition, we studied the known rs999737, rs2588809, and rs1314913 SNPs and *RAD51B* haplotypes in 44,791 breast cancer cases and 43,583 controls from 40 studies participating in the Breast Cancer Association Consortium (BCAC) that were genotyped on a custom chip (iCOGS). We identified one putatively pathogenic missense mutation c.541C>T among the Finnish cancer patients and subsequently genotyped the mutation in additional breast cancer cases (n = 5259) and population controls (n = 3586) from Finland and Belarus. No significant association with breast cancer risk was seen in the meta-analysis of the Finnish datasets or in the large BCAC dataset. The association with previously identified risk variants rs999737, rs2588809, and rs1314913 was replicated among all breast cancer cases and also among familial cases in the BCAC dataset. The most significant association was observed for the haplotype carrying the risk-alleles of all the three SNPs both among all cases (odds ratio (OR): 1.15, 95% confidence interval (CI): 1.11–1.19, *P* = 8.88 x 10^−16^) and among familial cases (OR: 1.24, 95% CI: 1.16–1.32, *P* = 6.19 x 10^−11^), compared to the haplotype with the respective protective alleles. Our results suggest that loss-of-function mutations in *RAD51B* are rare, but common variation at the *RAD51B* region is significantly associated with familial breast cancer risk.

## Introduction

Breast cancer is the most frequent cancer among women worldwide and also the leading cause of cancer-related death [1]. Several susceptibility loci for breast cancer have been identified and most of the currently known high- and moderate-penetrance predisposition genes have a role in DNA repair. The major high-penetrance breast and ovarian cancer susceptibility genes *BRCA1* and *BRCA2* are important for DNA double-strand break (DSB) repair through homologous recombination (HR) [2]. Proteins encoded by the moderate-penetrance genes *ATM*, *BRIP1*, and *CHEK2* interact with BRCA1 in DNA damage repair whereas PALB2 associates with both BRCA1 and BRCA2 [2,3]. In the HR repair of DSBs, the RAD51 recombinase has a key role. Binding of RAD51 to single-stranded DNA at the break site initiates the repair of a DSB [4]. In humans, there are five RAD51 paralogs RAD51B, RAD51C, RAD51D, XRCC2, and XRCC3, and they promote the binding of RAD51 to the DNA. Rare pathogenic mutations in *RAD51* paralogs *RAD51C* and *RAD51D* have been identified in breast and ovarian cancer families and confer a high risk specifically for ovarian cancer [5–7] whereas a homozygous missense mutation in *RAD51C* (*FANCO*) was found in a Fanconi anemia patient [8]. A homozygous *XRCC2* mutation has also been detected in a Fanconi anemia patient [9] and rare mutations in the gene in breast cancer families were identified in an exome sequencing study [10]; however, the association of *XRCC2* with breast cancer risk could not be confirmed in a large follow-up study [11].

Variants in the *RAD51B* region, also known as *RAD51L1*, have been associated with breast cancer risk in genome-wide association studies (GWAS) [12–14]. The major-allele of the common polymorphism rs999737 in intron 10 of *RAD51B* and the minor-allele of rs2588809 in intron 7 have been associated with an increased risk of female breast cancer [12,14]. The rs999737 has also been associated with breast cancer risk among *BRCA1* mutation carriers whereas no association was found for rs999737 or rs2588809 with breast cancer subtypes among *BRCA1* or *BRCA2* mutation carriers [15,16]. Another common polymorphism located in the intron 7 of the gene, rs1314913, has been associated with the risk of male breast cancer [13]. *RAD51B* is located at 14q24.1 and is expressed widely, with the highest levels in tissues that are active in recombination [17]. The RAD51B and RAD51C proteins form a stable complex which interacts weakly with RAD51 and promotes the assembly of RAD51 nuclear foci [18,19]. Furthermore, RAD51B is part of a larger BCDX2 complex that is formed with RAD51C, RAD51D, and XRCC2 [4]. The BCDX2 complex acts upstream of RAD51 recruitment to DNA damage foci [20]. Haploinsufficiency of *RAD51B* leads to aberrant HR repair of DNA and causes centrosome fragmentation and aneuploidy in human cells which suggests that loss of the proper biallelic expression of *RAD51B* may lead to chromosome instability in tumor cells [21].

In the Finnish population, recurrent founder mutations have been observed in many of the known breast and ovarian cancer susceptibility genes, including the RAD51 paralogs *RAD51C* and *RAD51D* [22,23]. Thus, the Finnish population is a valuable resource for the identification of new susceptibility alleles.

To identify putative recurrent founder mutations and to study the role of *RAD51B* in female and male breast cancer predisposition and especially in familial breast cancer, we comprehensively screened the *RAD51B* gene in 172 Finnish cancer patients. One identified missense mutation was *in silico* predicted to be pathogenic and was subsequently screened in a larger set of breast cancer cases and population controls from four datasets. In addition, the known *RAD51B* risk SNPs rs999737, rs2588809, and rs1314913 and *RAD51B* haplotypes were studied in the previously published 40 studies participating in the Breast Cancer Association Consortium (BCAC) including 44,791 breast cancer cases and 43,583 controls.

## Materials and Methods

### Screening of the *RAD51B* gene

The coding region and the exon-intron boundaries of the *RAD51B* gene (RefSeq NG_023267.1, NM_133509.3) were screened in 172 cancer patients from Southern Finland: in 87 female breast cancer patients with a family history of female breast cancer or breast and ovarian cancer, in 4 familial ovarian cancer patients, and in 4 female breast cancer patients with a family history of male and female breast cancer from the Helsinki region of Finland as well as in 77 male breast cancer patients (33 from the Helsinki region and 44 from the Tampere region) (S1 Text). All the female breast or ovarian cancer families and 60 of the male breast cancer families and patients were previously screened negative for *BRCA1/2* mutations whereas 21 patients had not been tested for *BRCA1/2* mutations. Genomic DNA isolated from blood was amplified by PCR and subsequently sequenced using ABI BigDyeTerminator 3.1 Cycle Sequencing kit (Life Technologies) (S1 Table). The sequencing results were analyzed with Variant Reporter Software v1.0 (Life Technologies).

### Genotyping of c.541C>T

The identified c.541C>T, p.(Arg181Trp) missense change was genotyped in additional familial and unselected breast cancer patients and population controls from the Helsinki (cases = 2203, controls = 1278), Tampere (cases = 704, controls = 800), and Oulu (cases = 452, controls = 273) regions of Finland and in 1900 cases and 1235 controls from Belarus (S1 Text). The Helsinki and Tampere datasets were genotyped by sequencing the exon 6 and the Oulu and Belarus datasets by high-resolution-melting (HRM) analysis (S1 Table). Written informed consent was obtained from all the participants and the study was approved by the Ethics Committees of Helsinki University Hospital, Tampere University Hospital, Oulu University Hospital, and by the institutional Ethics Commissions at the Minsk Mother and Child Hospital and at Hannover Medical School.

### iCOGS genotyping

The common *RAD51B* polymorphisms rs2588809, rs1314913, and rs999737 were studied in 44,791 invasive breast cancer cases and 43,583 controls from 40 studies (including partially the Helsinki, Oulu, and Belarus studies) participating in the Breast Cancer Association Consortium (BCAC) (S2 Table) [14]. The SNPs were genotyped on the iCOGS array as part of the Collaborative Oncological Gene-environment Study (COGS) as previously described [14] and genotypes for the c.541C>T missense were imputed with SHAPEIT and IMPUTEv2 by using the 1000Genomes project as the reference panel [24]. The analyses were restricted to cases with European ancestry. All participants gave written informed consent and all the studies were approved by the respective Institutional Review Boards or Ethics Committees (S2 Table).

### Bioinformatics and statistical methods

The pathogenicity of the variants identified in the sequencing of *RAD51B* was predicted using the MutationTaster software as it considers both exonic and intronic variants [25]. In addition, PON-P that utilizes results from SIFT, PhD-SNP, PolyPhen-2, SNAP, and I-Mutant 3 was used to predict the pathogenicity of the missense variants [26]. Secondary structure prediction was done with RaptorX [27] and protein-protein interaction with PredictProtein [28]. Statistical analyses were performed in R version 3.0.2 (http://www.r-project.org/). To study the association of the c.541C>T mutation with breast cancer risk, two-sided *P*-values were calculated using Pearson’s chi-squared test or, if the expected number of cell count was less than five, Fisher’s exact test. For meta-analysis, the estimates were combined using a fixed-effects meta-analysis, using the inverse variance-weighted method. Student’s t-test was used to compare the age at diagnosis between the mutation carriers and non-carriers. Per-allele odds ratios (OR) and confidence intervals (CI) for the common *RAD51B* polymorphisms were estimated with logistic regression. Multivariate regression models including any two of the three SNPs at a time were used to study the independence of the association signals. Haplotype-specific ORs and CIs were estimated with the HaploStats package in R. A haplotype carrying the major-alleles of rs2588809 and rs1314913 and the minor-allele of the rs999737 was used as a reference. Study and principal components were used as covariates in all analyses with the BCAC dataset to correct for potential population stratification. The analyses for the genotyped SNPs were adjusted for seven and the analysis for the imputed SNP for nine principal components as previously described [14,24]. Separate analyses were performed for all breast cancer cases and for subsets of cases with first-degree family history of breast cancer, and cases with estrogen receptor (ER) positive and negative tumors. Studies where family history information was predominantly missing were excluded from the familial analyses. The imputed c.541C>T missense variant was analyzed with SNPTEST. First, an association test stratified with study was performed to obtain imputation information scores for the individual studies. The final analysis, restricted to BCAC studies with information score ≥ 0.5, was performed with study and the nine principal components as covariates. The online tool LocusZoom was used to generate a regional association plot for visualization of the SNP associations and the extent of linkage disequilibrium (LD) [29].

## Results

We sequenced the coding region and the exon-intron boundaries of the *RAD51B* gene in 168 breast (female and male) and 4 familial ovarian cancer patients from Southern Finland. Nine intronic and six missense variants were identified (Table 1). The c.541C>T, p.(Arg181Trp), missense change was the only variant that was predicted to be pathogenic by both MutationTaster and PON-P software and was selected for further genotyping. Based on Finnish subjects in the ExAC dataset, the minor-allele frequency (MAF) for the c.541C>T variant is estimated to be 1.2% whereas in the non-Finnish Europeans the MAF is 0.05% (Exome Aggregation Consortium (ExAC), Cambridge, MA (URL: http://exac.broadinstitute.org) [May, 2015]). According to RaptorX secondary structure prediction software the arginine in position 181 is located in beta-sheet with the likelihood of 83.4% but, as determined by PredictProtein, the amino acid is not predicted to directly participate in protein-protein interactions.

**Table 1:** Variants identified in the screening of the *RAD51B* gene (RefSeq NM_133509.3).

DNA change	protein change	rs-number	position	AA ^a^	Aa ^b^	Aa ^c^	MAF ^d^	MAF_ExAC_ ^e^	MutationTaster	PON-P
c.84+28T>G		rs17783124	intron 2	71	84	17	0.343	0.287	polymorphism	
c.84+82T>C		rs28604984	intron 2	71	84	17	0.343		polymorphism	
c.84+120G>A		rs28623567	intron 2	71	84	17	0.343		polymorphism	
c.199-33G>T		rs184815928	intron 3	170	2	0	0.006	0.007	polymorphism	
c.199-30dupA		rs34564590	intron 3	70	85	17	0.346	0.286	polymorphism	
c.199-21C>T		rs35183950	intron 3	171	1	0	0.003	0.002	polymorphism	
c.515T>G	p.Leu172Trp	rs34094401	exon 6	167	5	0	0.015	0.027	polymorphism	unclassified
c.539A>G	p.Tyr180Cys	rs28910275	exon 6	145	27	0	0.078	0.057	polymorphism	unclassified
c.541C>T	p.Arg181Trp	rs199981178	exon 6	169	3	0	0.009	0.012	disease causing	pathogenic
c.572+95_96delGG			intron 6	171	1	0	0.003		polymorphism	
c.728A>G	p.Lys243Arg	rs34594234	exon 7	167	4	1	0.017	0.008	disease causing	unclassified
c.854-103A>G		rs10146321	intron 8	104	64	4	0.209		polymorphism	
c.957+125C>A		rs10146772	intron 9	48	83	41	0.480		polymorphism	
c.1063G>A	p.Ala355Thr	rs61758785	exon 11	170	2	0	0.006	0.007	polymorphism	neutral
c.1094C>G	p.Pro365Arg	rs28908468	exon 11	140	32	0	0.093	0.115	polymorphism	NA

Counts of ^**a**^ common homozygotes

^**b**^ heterozygotes and

^**c**^ rare homozygotes in this study

^**d**^ Minor-allele frequency (MAF) in this study

^**e**^ MAF in the Finnish population in the ExAC database

The c.541C>T missense variant was genotyped in additional 3359 female and male breast cancer patients and families, and in 2351 population controls from Southern (Helsinki and Tampere datasets) and Northern Finland (Oulu dataset). Altogether, when combining the two stages of mutation testing, c.541C>T was screened in 2331 patients from Helsinki, 748 patients from Tampere, and 452 patients from Oulu. There was no evidence of association in any of the population-based case-control studies (Table 2). There was a suggestive association among breast cancer families with at least three first or second-degree relatives affected with breast or ovarian cancer, compared to population controls, in the Helsinki dataset (OR: 2.31, 95% CI: 1.20–4.48, *P* = 0.010) (Table 2). The mean age at breast cancer diagnosis was similar between carriers and non-carriers among the familial patients (54.2 for carriers and 53.1 for non-carriers, *P* = 0.725) and among all cases (55.9 for carriers and 56.4 for non-carriers, *P* = 0.744). However, no association was observed for familial breast cancer in the Oulu dataset, and there was no evidence of association when all datasets were combined (OR: 1.35, 95% CI: 0.66–2.73, *P* = 0.410). The variant was also genotyped in 1900 breast cancer cases and 1235 population controls from Belarus but only three carriers were identified among cases and none among controls (Table 2).

**Table 2:** Frequencies and ORs for the c.541C>T mutation among the different patient subgroups in the Helsinki, Tampere, Oulu, and Belarus series.

**HELSINKI dataset**	**Total**	**CC**	**%**	**CT**	**%**	**OR (95% CI)**	***P***
Controls	1278	1257	98.36%	21	1.64%		
All female BC cases	2293	2240	97.69%	53	2.31%	1.42 (0.85–2.36)	0.179
Familial BC (≥3 affected)^a^	430	414	96.28%	16	3.72%	2.31 (1.20–4.48)	0.010
Familial BC (2 affected) ^b^	523	513	98.09%	10	1.91%	1.17 (0.55–2.50)	0.690
Unselected BC	1728	1690	97.80%	38	2.20%	1.35 (0.79–2.30)	0.277
Male BC ^c^	37	36	97.30%	1	2.70%	1.66 (0.22–12.70)	0.469
Familial OC	4	4	100%	0	0%	-	1
**TAMPERE dataset**	**Total**	**CC**	**%**	**CT/TT** ^d^	**%**	**OR (95% CI)**	***P***
Controls	800	778	97.25%	22	2.75%		
Unselected BC	645	620	96.12%	25	3.88%	1.43 (0.80–2.55)	0.230
Male BC ^e^	103	102	99.03%	1	0.97%	0.35 (0.05–2.60)	0.503
**OULU dataset**	**Total**	**CC**	**%**	**CT**	**%**	**OR (95% CI)**	***P***
Controls	273	267	97.80%	6	2.20%		
All female BC cases	452	443	98.01%	9	1.99%	0.90 (0.32–2.57)	0.850
Familial BC (≥3 affected)^a^	83	82	98.80%	1	1.20%	0.54 (0.06–4.57)	1
Familial BC (2 affected) ^b^	49	48	97.96%	1	2.04%	0.93 (0.11–7.87)	1
Unselected BC	320	313	97.81%	7	2.19%	1.00 (0.33–3.00)	0.993
**BELARUS dataset**	**Total**	**CC**	**%**	**CT**	**%**	**OR (95% CI)**	***P***
Controls	1235	1235	100%	0	0%	-	
BC cases	1900	1897	99.85%	3	0.15%	-	0.284

BC = breast cancer; OC = ovarian cancer

^a^ Families with ≥3 BC or OC among first- or second-degree relatives

^b^ Two first-degree relatives affected with BC or OC

^c^ Includes 33 male BC cases and 4 female BC cases with family history of male BC

^d^ The Tampere dataset included one case with homozygous mutation

^e^ Male BC cases only

We further studied the missense variant c.541C>T and the previously reported common risk SNPs rs2588809, rs1314913, and rs999737 in a large breast cancer dataset from BCAC comprising 40 studies with 44,791 invasive breast cancer cases and 43,583 controls of predominantly European ancestry. The subjects were genotyped on an Illumina Infinium custom chip (iCOGS) for over 200,000 SNPs [14], including the rs2588809, rs1314913, and rs999737 SNPs. Genotypes for over 11 million SNPs, including the c.541C>T missense variant, were imputed by using the 1000Genomes project as a reference panel [24]. For the missense variant, we first performed an association test stratified by study including all the 40 BCAC studies. The overall information score for the c.541C>T was 0.673. To increase the imputation accuracy of this rare variant, studies with information score less than 0.5 in the stratified analysis were excluded from the final analysis (S3 Table). Altogether 26,969 cases and 27,092 controls from 23 studies were included and the information score for the variant in the final analysis was 0.755. The MAF of the c.541C>T missense variant was 0.2% among all cases, familial cases, and controls and it did not associate with breast cancer among all cases (OR: 0.97, 95% CI: 0.76–1.25, *P* = 0.538) or familial cases (OR: 1.04, 95% CI: 0.65–1.66, *P* = 0.794). The highest frequency was observed in the Finnish studies HEBCS, KBCP, and OBCS with the overall MAF ranging from 0.6% to 0.7%. The HEBCS and OBCS studies are overlapping with the Finnish Helsinki and Oulu datasets that were genotyped for the variant.

Breast cancer associations for two of the genotyped SNPs, rs2588809 and rs999737, in the BCAC dataset have been published before [14,24,30] and the results were replicated here. The minor-alleles of rs2588809 and rs1314913 and the major-allele of rs999737 were associated with an increased risk of breast cancer with ORs between 1.07–1.08 among all breast cancer cases and also among familial cases with ORs between 1.10–1.15 (Table 3). The associations were also significant among the subset of cases with ER-positive tumors but only rs999737 showed association among the ER-negative subset (Table 3). Rs2588809 and rs1314913 are strongly correlated (r^2^ = 0.816), whereas rs999737 is not correlated with them (r^2^ = 0.003 with rs2588809 and r^2^ = 0.070 with rs1314913) (S1 Fig). In multivariate logistic regression models, both rs2588809 and rs1314913 remained significant after adjustment with rs999737 (OR: 1.07, 95% CI: 1.04–1.09, *P* = 1.74 x 10^−6^ and OR: 1.06, 95% CI: 1.03–1.09, *P* = 9.24 x 10^−6^, respectively). Likewise, rs999737 remained an independent predictor for risk after adjustment with rs2588809 or rs1314913 (OR: 1.08, 95% CI: 1.06–1.11, *P* = 2.91 x 10^-11^and *P* = 2.54 x 10^−11^, respectively). Neither rs2588809 nor rs1314913 remained significant when adjusted with each other (OR: 1.07, 95% CI: 1.00–1.16, *P* = 0.066 and OR: 1.00, 95% CI: 0.93–1.08, *P* = 0.977, respectively).

**Table 3:** Frequencies and ORs for the SNPs rs2588809, rs1314913, and rs999737 for all breast cancer cases, familial cases and ER-positive and -negative cases in the BCAC dataset.

Rs-number	MAF _controls_	MAF _cases_	OR_ALL_ (95%CI)	*P*_ALL_	OR_FAM_ (95%CI)	*P*_FAM_	OR_ER+_ (95%CI)	*P*_ER+_	OR_ER-_ (95%CI)	*P*_ER-_
rs2588809	0.158	0.169	1.08 (1.05–1.10)	4.62 x 10^−8^	1.10 (1.05–1.16)	9.72 x 10^−5^	1.09 (1.06–1.12)	1.65 x 10^−8^	1.00 (0.95–1.05)	8.90 x 10^−1^
rs1314913	0.144	0.154	1.07 (1.04–1.10)	2.74 x 10^−7^	1.10 (1.04–1.16)	2.45 x 10^−4^	1.09 (1.05–1.12)	1.36 x 10^−7^	0.98 (0.93–1.03)	3.85 x 10^−1^
rs999737	0.231	0.215	1.09 (1.06–1.11)	7.78 x 10^−13^	1.15 (1.10–1.20)	1.08 x 10^−9^	1.10 (1.07–1.13)	7.70 x 10^−12^	1.05 (1.00–1.10)	3.03 x 10^−2^

ALL = all breast cancer cases; FAM = familial breast cancer cases; ER+ = ER-positive; ER- = ER- negative.

To evaluate the allele combination associated with the highest risk, we performed haplotype analysis for rs2588809, rs1314913, and rs999737. The reference haplotype CCT, carrying the major-alleles of rs2588809 and rs1314913 and the minor-allele of rs999737 had an estimated frequency of 21.0% among controls and 19.4% among cases. The strongest association was observed for the TTC haplotype that carries the risk-alleles of all the three SNPs with an odds ratio of 1.15 for all cases (95% CI: 1.11–1.19, *P* = 8.88 x 10^−16^) and 1.24 for familial cases (95% CI: 1.16–1.32, *P* = 6.19 x 10^−11^) (Table 4). This haplotype had an estimated frequency of 12.6% among controls, 13.5% among all cases, and 14.1% among familial cases. The most common haplotype, with over 60% frequency among cases and controls, was the haplotype CCC carrying the risk-allele of rs999737 and also this haplotype associated with an increased risk of breast cancer among all the cases (OR: 1.09, 95% CI: 1.06–1.12, *P* = 1.86 x 10^−10^) and the familial cases (OR: 1.14, 95% CI: 1.09–1.20, *P* = 1.30 x 10^−7^) when compared to the CCT haplotype.

**Table 4:** Estimated haplotype frequencies and haplotype-specific ORs among the BCAC dataset. The haplotypes are based on genotypes for SNPs rs2588809, rs1314913, and rs999737.

	All breast cancer cases				Familial breast cancer cases			
Haplotype	Freq controls	Freq cases	OR (95% CI)	*P*	Freq controls	Freq cases	OR (95% CI)	*P*
CCT (reference)	0.210	0.194			0.211	0.188		
TTC	0.126	0.135	1.15 (1.11–1.19)	8.88 x 10^−16^	0.126	0.141	1.24 (1.16–1.32)	6.19 x 10^−11^
CCC	0.632	0.636	1.09 (1.06–1.12)	1.86 x 10^−10^	0.628	0.635	1.14 (1.09–1.20)	1.30 x 10^−7^
TCC	0.011	0.013	1.15 (1.04–1.27)	5.48 x 10^−3^	0.012	0.013	1.17 (0.98–1.41)	8.36 x 10^−2^
TTT	0.018	0.018	1.11 (0.99–1.23)	7.12 x 10^−2^	0.018	0.017	1.*06* (0.86–1.31)	5.70 x 10^−1^

Freq = frequency

## Discussion

To study the role of *RAD51B* in breast cancer predisposition, we screened the coding sequence in 172 Finnish breast or ovarian cancer patients. We also studied the common susceptibility variants in the genomic region of the gene in a large dataset from BCAC including 44,791 breast cancer cases and 43,583 controls. Among the Finnish patients, we identified one putatively pathogenic missense variant, c.541C>T, p.(Arg181Trp), that was further screened in a larger set of unselected and familial breast cancer patients and population controls.

In the Helsinki dataset, the c.541C>T mutation had a suggestive association with familial breast cancer but this association was not confirmed in the other Finnish sample sets. In Belarus, the mutation was rare and was identified only in three cases but not among controls. For the large BCAC dataset that had been genotyped on the iCOGS chip, c.541C>T genotypes were imputed. The missense was very rare with a 0.2% MAF and no association with breast cancer was seen among all breast cancer cases or in the subset of familial patients.

Out of the common variants at the *RAD51B* region, rs999737 and rs2588809 have been associated with female breast cancer and rs1314913 with male breast cancer [12–14,30]. For rs999737 and rs2588809 the results from the previous BCAC studies for all cases and by ER status were replicated here [14,30]. The rs1314913 SNP has been previously associated with male breast cancer with an OR of 1.57 (*P* = 3.02 × 10^−13^) [13]. It has not been previously reported in female breast cancer, and showed here similar associations as rs2588809 with OR of 1.07 among all cases and 1.09 among ER-positive cases. The rs1314913 SNP is correlated with rs2588809 (r^2^ = 0.816) but not with rs999737 (r^2^ = 0.070). Our results suggest two independent associations at the region as rs999737 adjusted with rs1314913 or rs2588809 showed significant association whereas neither rs1314913 nor rs2588809 remained significant after adjustment with each other, suggesting both may be tagging another, causative allele.

All three variants were also associated with familial breast cancer. The strongest association was observed for a haplotype carrying the risk-alleles of all the three SNPs both among all cases and familial cases with ORs of 1.15 and 1.24, respectively. This haplotype is observed at a frequency of 12.6% among controls and 13.5% among cases.

To date, only three studies have reported truncating germline mutations in *RAD51B*: two deleterious mutations were detected among ovarian cancer cases, one splicing mutation in a breast and ovarian cancer family, and one nonsense mutation in a melanoma family [31–33]. In an Australian, study no pathogenic mutations were identified among 188 breast cancer families [34]. These reports together with our results suggest that loss-of-function mutations in *RAD51B* are very rare yet we cannot exclude such mutations and possibly a higher breast cancer risk associated with these. However, the common variants in the *RAD51B* region are associated with an increased breast cancer risk. Further fine-mapping studies are needed to identify the causative variants underlying the associations, and functional studies to determine whether *RAD51B* or another gene in the region is the target of these associations. *RAD51B* is a plausible candidate gene for the association as it functions in HR repair of DNA damage like most of the known breast cancer genes.

In conclusion, no pathogenic *RAD51B* mutations were identified among 172 Finnish breast or ovarian cancer patients. However, we cannot rule out rare risk-variants in the Finnish or other populations. The minor-alleles of the common polymorphisms rs2588809 and rs1314913 and the major-allele of rs999737 were associated with breast cancer risk in the large BCAC dataset. The strongest association was observed for a risk haplotype carrying the risk-alleles of all the three SNPs with an OR of 1.15 among all cases and 1.24 among familial cases compared to the haplotype with the respective protective alleles.

## Supporting Information

S1 Fig Regional association plot for the *RAD51B* SNPs rs2588809, rs1314913, and rs999737 and the c.541C>T missense variant. Each variant is represented with a dot and the color of the dot represents the extent of LD (r^2^) with rs2588809. Association among all breast cancer cases in the BCAC dataset is represented at the–log10 scale. The *x* axis shows the genomic positions of the variants based on hg19 build. The right *y* axis shows the estimated recombination rate at the region (centiMorgans/megabase, cM/Mb). LD and recombination rate were estimated using the 1000Genomes Nov 2014 EUR as the reference population.
(PDF)

S1 Table Primers and PCR conditions for the screening of the *RAD51B* gene and the c.541C>T missense mutation. (DOCX)

S2 Table Description of the BCAC studies. BCAC studies genotyped on the iCOGS array, ethics approval committees/institutional review boards and the numbers of controls, all invasive cases, cases with first-degree family history of breast cancer, and cases with ER-positive and negative tumors included in this study. All subjects are genetically European.
(DOCX)

S3 Table The imputed c.541C>T variant in the BCAC dataset. Minor-allele frequencies (MAF) of the imputed c.541C>T missense variant in the BCAC studies and the information scores from the association analysis stratified by study. Studies with information score <0.5 were excluded from the final analysis.
(DOCX)

S1 Text Supplementary Materials and Methods. (DOCX)
