# Supplementary figures and images for: RAD51B in Familial Breast Cancer
Source: PLoS One. 2016 May 5;11(5):e0153788. doi: 10.1371/journal.pone.0153788 (PMC4858276; doi:10.1371/journal.pone.0153788)

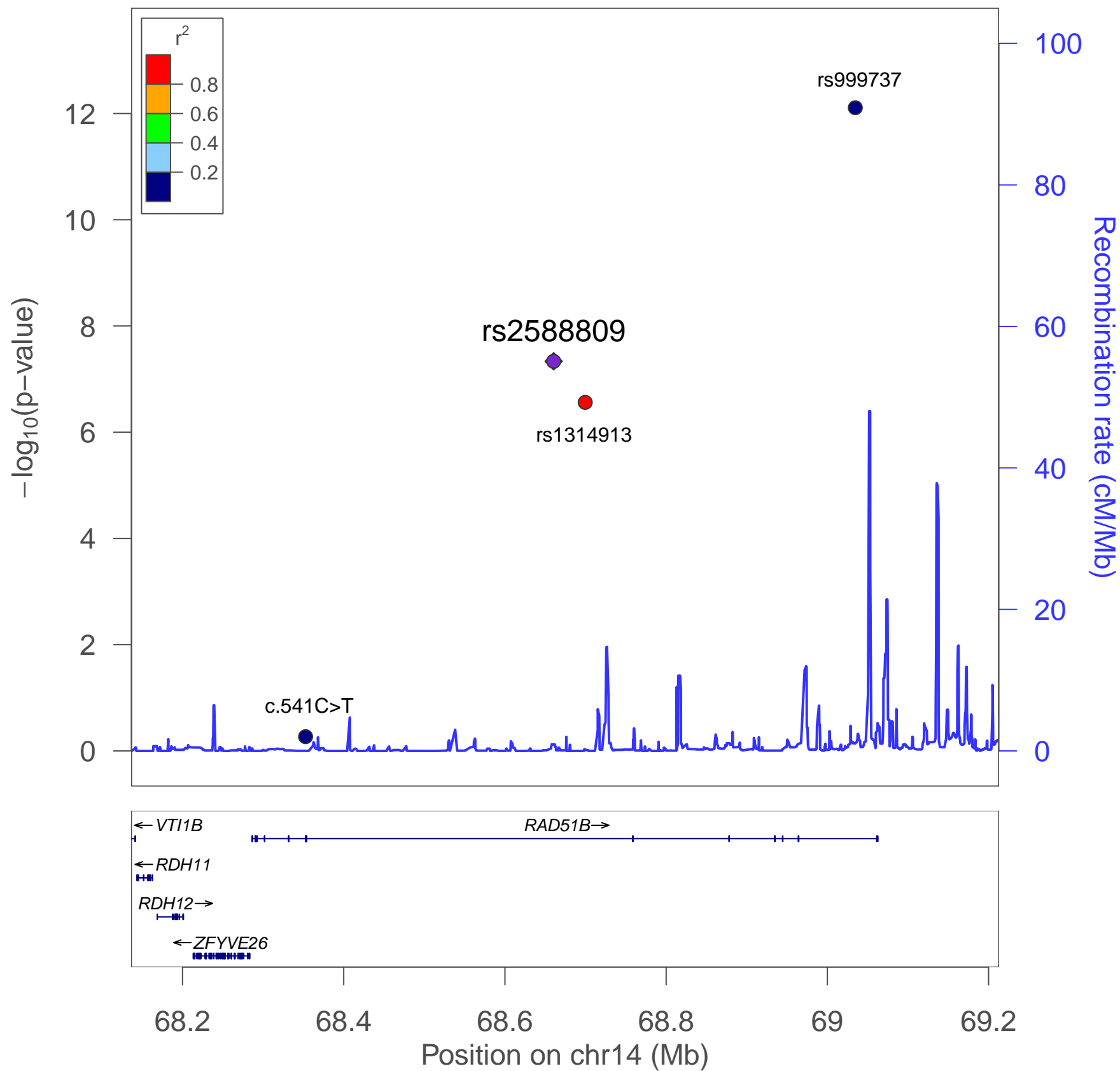

Supplement: S1 Fig — Each variant is represented with a dot and the color of the dot represents the extent of LD (r2) with rs2588809. Association among all breast cancer cases in the BCAC dataset is represented at the–log10 scale. The x axis shows the genomic positions of the variants based on hg19 build. The right y axis shows the estimated recombination rate at the region (centiMorgans/megabase, cM/Mb). LD and recombination rate were estimated using the 1000Genomes Nov 2014 EUR as the reference population. (PDF) [file pone.0153788.s001.pdf]
