# Supplementary material for: RAD51B in Familial Breast Cancer
Source: PLoS One. 2016 May 5;11(5):e0153788. doi: 10.1371/journal.pone.0153788 (PMC4858276; doi:10.1371/journal.pone.0153788)
Supplement: S1 Table — (DOCX) [file pone.0153788.s002.docx]

**S1 Table. Primers and PCR conditions for the screening of the *RAD51B* gene and the c.541C>T missense mutation.**

| **Amplicon** | **bp** | **Forward primer (5´….3´)** | **Reverse primer (5´….3´)** | **Temp (C°)** |
| --- | --- | --- | --- | --- |
| ex1 | 184 | CGGCCTCTGATTGGATACAG | TAATGAGCGAACCCCACTTC | 63 |
| ex2 | 366 | TTGCATATGGAGAAACTTGAGG | CACCCAAGGAGGCTTGATATT | 57 |
| ex3 | 282 | AAGCAGTATCAATTGAAGGTTTCC | GCATGTGCCTCATCCCTAAA | 58 |
| ex4 | 374 | GGGAAAAGGATGGATTAGGG | TGAGTGGAGAACCATATCCTTG | 57 |
| ex5 | 340 | CTGGGAACCCAGGTTAGTTG | CCTATGTGTAAATGTTTCATGCTGTA | 58 |
| ex6 | 352 | GAGATTCAGCAATGTGGCTTT | CAAGAAAGACTTCAACCAAAAGG | 55 |
| ex6_tumor | 177 | TTTTCCCAGATATTTTAACACTGA | TTCACTTAGAAGCTGAAACATAAAAGA | 55 |
| ex6_HRM | 102 | TCCCAGATATTTTAACACTGAAGAAA | GCAGCATACCTTTGTAGAACTTCAT | 58 |
| ex7 | 378 | TGCTTAGCCTTTGCAATACCTT | AAGTCATCTGTTTTACATTTTCCTCT | 55 |
| ex8 | 284 | GTCTTCTCCTAAACCATAGTCTGC | AGCTCTCAACAGGAGGTGGA | 63 |
| ex9 | 476 | CTTTCACCAAGGCTGAAGGA | CTGTTGTTGGGACAAGCAGA | 58 |
| ex10 | 288 | AAGTGGGAAGGGGAGTGAATA | ATCACTGGCCAATGCATCTT | 63 |
| ex11 | 452 | TTCCTATGCCATACCCCTTG | ATTGCCCCCTTTCTCATTCT | 58 |

bp = amplicon length in base pairs, Temp = annealing temperature;

ex6_tumor primers were used to analyse the tumor DNA samples of male breast cancer patients from Tampere and ex6_HRM primers were used in the high-resolution-melting (HRM) analysis.
