# Supplementary material for: RAD51B in Familial Breast Cancer
Source: PLoS One. 2016 May 5;11(5):e0153788. doi: 10.1371/journal.pone.0153788 (PMC4858276; doi:10.1371/journal.pone.0153788)
Supplement: S2 Table — BCAC studies genotyped on the iCOGS array, ethics approval committees/institutional review boards and the numbers of controls, all invasive cases, cases with first-degree family history of breast cancer, and cases with ER-positive and negative tumors included in this study. All subjects are genetically European. (DOCX) [file pone.0153788.s003.docx]

**S2 Table. Description of the BCAC studies.** BCAC studies genotyped on the iCOGS array, ethics approval committees/institutional review boards and the numbers of controls, all invasive cases, cases with first-degree family history of breast cancer, and cases with ER-positive and negative tumors included in this study. All subjects are genetically European.

| **Study** | **Country** | **Approval Committee** | **Controls**  **n** | **Invasive cases**  **n** | **Familial cases**  **n** | **ER+ cases**  **n** | **ER- cases**  **n** |
| --- | --- | --- | --- | --- | --- | --- | --- |
| ABCFS | Australia | The University of Melbourne Health Sciences Human Ethics Sub-Committee (HESC) | 551 | 790 | 141 | 456 | 261 |
| ABCS | Netherlands | Leiden University Medical Center (LUMC) Commissie Medische Ethiek and Protocol Toetsingscommissie van het Nederlands Kanker Instituut/Antoni van Leeuwenhoek Ziekenhuis | 1815 | 1991 | 390 | 780 | 282 |
| BBCC | Germany | Friedrich-Alexander-Universitat Erlangen-Nurnberg Medizinische Fakultat Ethik-Commission | 458 | 554 | 78 | 456 | 82 |
| BBCS | UK | South East Multi-Centre Research Ethics Committee | 1397 | 1446 | 1127 | 507 | 113 |
| BIGGS | Ireland | Galway University College Hospital Clinical Research Ethical Committee | 719 | 793 | 94 | 474 | 146 |
| BSUCH | Germany | Medizinische Fakultat Heidelberg Ethikkommission | 954 | 815 | 50 | 531 | 152 |
| CECILE | France | Comite Consultatif de Protection des Personnes dans la Recherche Biomedicale de Bicetre | 999 | 900 | 153 | 743 | 130 |
| CGPS | Denmark | Kobenhavns Amt den Videnskabsetiske Komite | 4534 | 2858 | 340 | 1919 | 357 |
| CNIO-BCS | Spain | Hospital Universitario La Paz Comite Etico de Investigacion Clinica | 876 | 877 | 195 | 225 | 81 |
| CTS | USA | UC Irvine: Office of Research Institutional Review Board | 71 | 68 | 7 | 0 | 68 |
| ESTHER | Germany | Ruprecht-Karls-Universitat Medizinische Fakultat Heidelberg Ethikkommission | 502 | 472 | 73 | 303 | 98 |
| GC-HBOC ^a^ | Germany | Ethik-Kommission der Medizinischen Fakultat der Universitat zu Koln | 139 | 0 | - | - | - |
| GENICA | Germany | Rheinische Friedrich-Wilhelms-Universitat Medizinische Einrichtungen Ethik-Kommission | 427 | 465 | 61 | 328 | 119 |
| HEBCS | Finland | Helsinki University Hospital Ethics Committee | 1234 | 1517 | 586 | 1235 | 216 |
| HMBCS | Belarus | Medizinische Hochschule Hannover Ethik-Kommission | 130 | 688 | - | 35 | 8 |
| **Study** | **Country** | **Approval Committee** | **Controls**  **n** | **Invasive cases**  **n** | **Familial cases**  **n** | **ER+ cases**  **n** | **ER- cases**  **n** |
| KARBAC | Sweden | Lokala Forskningsetikkommitten Nord | 662 | 722 | 275 | 338 | 63 |
| KBCP | Finland | The joint ethics committee of Kuopio University and Kuopio University Hospital | 251 | 411 | 45 | 288 | 89 |
| kConFab/AOCS | Australia | kConFab: The Queenland Institute of Medical Research Human Research Ethics Committee (QIMR-HREC) | 897 | 410 | 280 | 135 | 50 |
| LMBC | Belgium | Commissie Medische Ethiek van de Universitaire Ziekenhuizen Kuleuven | 1388 | 2616 | 423 | 2069 | 378 |
| MARIE | Germany | Ruprecht-Karls-Universitat Medizinische Fakultat Heidelberg Ethikkommission | 1778 | 1656 | 265 | 1279 | 370 |
| MBCSG | Italy | Comitato Etico Indipendente della Fondazione IRCCS "Istituto Nazionale dei Tumori" | 400 | 189 | 88 | 128 | 40 |
| MCBCS | USA | Mayo Clinic IRB | 1931 | 1546 | 326 | 1271 | 250 |
| MCCS | Australia | The Cancer Council Victoria Human Research Ethics Committee | 511 | 614 | - | 352 | 119 |
| MEC | USA | University of Southern California Health Sciences Campus IRB | 741 | 705 | 120 | 412 | 87 |
| MTLGEBCS | Canada | McGill University IRB and Research Ethics Committees of CSSS Dorval-Lachine-LaSalle, CHU Sainte-Justine, Centre Hospitalier de l'Universite de Montreal, CSSS Sud-Ouest-Verdun, CSSS d'Ahuntsic et Montreal-Nord, CSSS de l'Ouest-de-l'Ile , Centre Hospitalier Universitaire de Quebec, Hopital Maisonneuve-Rosemont, Hopital Santa Cabrini, and CSSS Coeur-de-l'Ile | 436 | 489 | 117 | 421 | 64 |
| NBCS | Norway | Regional Komite for Medisinsk Forskningsetikk (Helseregion III Universitetet I Bergen, Universitetet I Oslo, Helseregion Sor, Helseregion II, and Ost-Norge) | 217 | 860 | - | 617 | 199 |
| NBHS | USA | Vanderbilt University IRB | 118 | 125 | 25 | 0 | 125 |
| OBCS | Finland | Oulu University Hospital Ethics Committee | 414 | 500 | - | 403 | 97 |
| OFBCR | Canada | Mount Sinai Hospital Research Ethics Board | 511 | 1157 | 473 | 629 | 267 |
| ORIGO | Netherlands | Medical Ethical Committee and Board of Directors of the Leiden University Medical Center (LUMC) | 327 | 335 | 47 | 211 | 68 |
| **Study** | **Country** | **Approval Committee** | **Controls**  **n** | **Invasive cases**  **n** | **Familial cases**  **n** | **ER+ cases**  **n** | **ER- cases**  **n** |
| PBCS | Poland | National Institute of Health (NIH) IRB | 424 | 519 | 46 | 519 | 0 |
| pKARMA | Sweden | Regional Ethical Review Board in Stockholm | 5568 | 4553 | - | 3588 | 664 |
| RBCS | Netherlands | Medische Ethische Toetsings Commissie Erasmus Medisch Centrum | 699 | 623 | 318 | 367 | 125 |
| SASBAC | Sweden | Regional Ethical Review Board in Stockholm | 1378 | 1163 | 176 | 663 | 144 |
| SBCS | UK | South Sheffield Research Ethics Committee | 848 | 751 | 114 | 358 | 104 |
| SEARCH | UK | Multi Centre Research Ethics Committee (MREC) | 8069 | 9096 | 1239 | 5130 | 1170 |
| SKKDKFZS ^a^ | Germany | Ethics Commission of the Medical Faculty of Heidelberg | 0 | 134 | - | 0 | 134 |
| SZBCS | Poland | Komisji Bioetycznej Pomorskiej Akademii Medycznej | 315 | 303 | 30 | 149 | 51 |
| TNBCC | Various | Ethics Committees of National Centre of Scientific Research "Demokritos" and Aristotle University of Thessaloniki Medical School and Institutional Review Boards of OSU Cancer and RPCI | 424 | 667 | 44 | 0 | 667 |
| UKBGS | UK | South East Multi-Centre Research Ethics Committee | 470 | 413 | 33 | 0 | 0 |

^a^ Controls for SKKDKFZS were selected from GC-HBOC study
