# Supplementary material for: RAD51B in Familial Breast Cancer
Source: PLoS One. 2016 May 5;11(5):e0153788. doi: 10.1371/journal.pone.0153788 (PMC4858276; doi:10.1371/journal.pone.0153788)
Supplement: S3 Table — Minor-allele frequencies (MAF) of the imputed c.541C>T missense variant in the BCAC studies and the information scores from the association analysis stratified by study. Studies with information score <0.5 were excluded from the final analysis. (DOCX) [file pone.0153788.s004.docx]

**S3 Table.** **The imputed c.541C>T variant in the BCAC dataset**. Minor-allele frequencies (MAF) of the imputed c.541C>T missense variant in the BCAC studies and the information scores from the association analysis stratified by study. Studies with information score <0.5 were excluded from the final analysis.

| **Study** | **Country** | **Information score** | **Total MAF** | **MAF in controls** | **MAF in cases** | **Included in the final analysis** |
| --- | --- | --- | --- | --- | --- | --- |
| ABCFS | Australia | 0.526 | 0.002 | 0.001 | 0.002 | yes |
| ABCS | Netherlands | 0.389 | 0.001 | 0.001 | 0.001 | - |
| BBCC | Germany | 0.561 | 0.001 | 0.002 | 0.000 | yes |
| BBCS | UK | 0.573 | 0.002 | 0.002 | 0.002 | yes |
| BIGGS | Ireland | 0.556 | 0.002 | 0.002 | 0.002 | yes |
| BSUCH | Germany | 0.599 | 0.002 | 0.002 | 0.001 | yes |
| CECILE | France | 0.207 | 0.001 | 0.001 | 0.000 | - |
| CGPS | Denmark | 0.771 | 0.001 | 0.001 | 0.002 | yes |
| CNIO-BCS | Spain | 0.201 | 0.000 | 0.000 | 0.001 | - |
| CTS | USA | 0.996 | 0.004 | 0.007 | 0.000 | yes |
| ESTHER | Germany | 0.721 | 0.002 | 0.002 | 0.001 | yes |
| GENICA | Germany | 0.167 | 0.000 | 0.000 | 0.000 | - |
| HEBCS | Finland | 0.916 | 0.007 | 0.007 | 0.008 | yes |
| HMBCS | Belarus | 0.773 | 0.002 | 0.000 | 0.002 | yes |
| KARBAC | Sweden | 0.852 | 0.003 | 0.003 | 0.003 | yes |
| KBCP | Finland | 0.953 | 0.007 | 0.006 | 0.008 | yes |
| kConFab/AOCS | Australia | 0.547 | 0.001 | 0.001 | 0.001 | yes |
| LMBC | Belgium | 0.569 | 0.001 | 0.001 | 0.001 | yes |
| MARIE | Germany | 0.739 | 0.002 | 0.003 | 0.001 | yes |
| MBCSG | Italy | 0.164 | 0.000 | 0.000 | 0.001 | - |
| MCBCS | USA | 0.633 | 0.002 | 0.002 | 0.001 | yes |
| MCCS | Australia | 0.182 | 0.001 | 0.001 | 0.001 | - |
| MEC | USA | 0.446 | 0.001 | 0.001 | 0.001 | - |
| MTLGEBCS | Canada | 0.176 | 0.001 | 0.001 | 0.000 | - |
| NBCS | Norway | 0.595 | 0.001 | 0.001 | 0.001 | yes |
| NBHS | USA | 0.259 | 0.001 | 0.000 | 0.001 | - |
| OBCS | Finland | 0.858 | 0.006 | 0.009 | 0.004 | yes |
| OFBCR | Canada | 0.602 | 0.001 | 0.001 | 0.000 | yes |
| ORIGO | Netherlands | 0.169 | 0.000 | 0.000 | 0.000 | - |
| **Study** | **Country** | **Information score** | **Total MAF** | **MAF in controls** | **MAF in cases** | **Included in the final analysis** |
| PBCS | Poland | 0.414 | 0.001 | 0.002 | 0.001 | - |
| pKARMA | Sweden | 0.767 | 0.003 | 0.003 | 0.003 | yes |
| RBCS | Netherlands | 0.759 | 0.001 | 0.000 | 0.001 | yes |
| SASBAC | Sweden | 0.669 | 0.002 | 0.002 | 0.002 | yes |
| SBCS | UK | 0.796 | 0.001 | 0.001 | 0.002 | yes |
| SEARCH | UK | 0.480 | 0.002 | 0.002 | 0.002 | - |
| SKKDKFZS/GC-HBOC | Germany | 0.030 | 0.000 | 0.000 | 0.000 | - |
| SZBCS | Poland | 0.034 | 0.000 | 0.000 | 0.000 | - |
| TNBCC | Various | 0.284 | 0.001 | 0.001 | 0.001 | - |
| UKBGS | UK | 0.433 | 0.001 | 0.001 | 0.001 | - |
| **Total** |  | **0.673** | **0.002** | **0.002** | **0.002** |  |
