# Supplementary material for: RAD51B in Familial Breast Cancer
Source: PLoS One. 2016 May 5;11(5):e0153788. doi: 10.1371/journal.pone.0153788 (PMC4858276; doi:10.1371/journal.pone.0153788)
Supplement: S1 Text — (DOCX) [file pone.0153788.s005.docx]

**S1 Text. Supplementary Materials and Methods.**

**Helsinki dataset.** The unselected breast cancer patient series from Helsinki was collected at Helsinki University Central Hospital Department of Oncology in 1997-1998 and 2000 (884 patients, including 79% of all consecutive, newly diagnosed breast cancer cases during the collection periods) [1, 2] and Department of Surgery in 2001-2004 (986 patients, including 87% of all consecutive, newly diagnosed breast cancer cases) [3, 4]. A total of 1728 female patients with invasive breast cancer were included in the analysis for the *RAD51B* c.541C>T. The additional familial breast and ovarian cancer patients and one of the male breast cancer patients were collected at Helsinki University Hospital Departments of Oncology and Clinical Genetics. Three of the male breast cancer patients were identified as part of the unselected breast cancer series, and the additional male cases (n = 29) were diagnosed with breast cancer between 2004 and 2011 at Helsinki University Central Hospital Department of Oncology and the samples were collected in 2009-2012. Altogether 430 patients from Helsinki, tested negative for *BRCA1/2* mutations, had strong family history of breast cancer with at least three breast or ovarian cancer cases among the first- or second-degree relatives (including the proband), and 523 patients, tested negative for the Finnish *BRCA1/2* founder mutations, had one first-degree relative affected with breast or ovarian cancer [5-7]. The genealogies were confirmed through population registries and cancer diagnoses through the Finnish Cancer Registry and hospital records.

**Tampere dataset.** The unselected breast cancer series from Tampere was collected at Tampere University Hospital as previously described [1, 3], and additional 336 incident cases were collected in 1996-2004 at Tampere University Hospital. Only invasive cases were included in the analysis. The unselected series included three male breast cancer patients and one of them was included both in the unselected series and in male breast cancer series. The male breast cancer cases were identified as previously described [8] and genomic DNA was isolated from the blood (n = 44) and paraffin-embedded tumour samples (n = 59).

**Oulu dataset.** The unselected and the familial breast cancer samples from Oulu consisted of 320 and 132 patients, respectively, and the samples were collected at the Oulu University Hospital as previously described [9]. Of the familial patients, 83 had strong family history of breast cancer with at least three breast or ovarian cancer cases among first- or second-degree relatives, including the proband, and 49 breast cancer patients had one affected first-degree relative.

**Belarus dataset.** The series from Belarus consisted of 1900 breast cancer patients diagnosed in the Republic of Belarus during the years 1998–2008. Patients were recruited at the Byelorussian Institute for Oncology and Medical Radiology Aleksandrov N N. in Minsk or at one of five regional oncology centers in Gomel, Mogilev, Grodno, Brest or Vitebsk, as previously described [10]. The Belarus series mainly consisted of consecutive patients unselected for family history, with the exception of an additional 28 cases with familial breast cancer ascertained at the center in Minsk. Median age at diagnosis in the Belarus cohort was 48 years, and 305 patients (16%) reported a first-degree relative with breast cancer. Byelorussian population controls were 1235 healthy volunteers from the same population who had no personal history of breast cancer at the time when entering the study.

# References

1. Syrjäkoski K, Vahteristo P, Eerola H et al (2000) Population-based study of BRCA1 and BRCA2 mutations in 1035 unselected Finnish breast cancer patients. J Natl Cancer Inst 92:1529-1531

2. Kilpivaara O, Bartkova J, Eerola H et al (2005) Correlation of CHEK2 protein expression and c.1100delC mutation status with tumor characteristics among unselected breast cancer patients. Int J Cancer 113:575-580. doi: 10.1002/ijc.20638

3. Fagerholm R, Hofstetter B, Tommiska J et al (2008) NAD(P)H:quinone oxidoreductase 1 NQO1*2 genotype (P187S) is a strong prognostic and predictive factor in breast cancer. Nat Genet 40:844-853

4. Eerola H, Blomqvist C, Pukkala E, Pyrhonen S, Nevanlinna H (2000) Familial breast cancer in southern Finland: how prevalent are breast cancer families and can we trust the family history reported by patients? Eur J Cancer 36:1143-1148

5. Vehmanen P, Friedman LS, Eerola H et al (1997) Low proportion of BRCA1 and BRCA2 mutations in Finnish breast cancer families: evidence for additional susceptibility genes. Hum Mol Genet 6:2309-2315

6. Vahteristo P, Eerola H, Tamminen A, Blomqvist C, Nevanlinna H (2001) A probability model for predicting BRCA1 and BRCA2 mutations in breast and breast-ovarian cancer families. Br J Cancer 84:704-708. doi: 10.1054/bjoc.2000.1626

7. Vahteristo P, Bartkova J, Eerola H et al (2002) A CHEK2 genetic variant contributing to a substantial fraction of familial breast cancer. Am J Hum Genet 71:432-438. DOI:10.1086/341943

8. Syrjäkoski K, Hyytinen ER, Kuukasjärvi T, Auvinen A, Kallioniemi OP, Kainu T, Koivisto PA (2003) Androgen receptor gene alterations in Finnish male breast cancer. Breast Cancer Res Treat 77:167-170

9. Vuorela M, Pylkäs K, Hartikainen JM et al (2011) Further evidence for the contribution of the RAD51C gene in hereditary breast and ovarian cancer susceptibility. Breast Cancer Res Treat 130:1003-1010. doi: 10.1007/s10549-011-1677-x

10. Bogdanova NV, Antonenkova NN, Rogov YI, Karstens JH, Hillemanns P, Dörk T (2010) High frequency and allele-specific differences of BRCA1 founder mutations in breast cancer and ovarian cancer patients from Belarus. Clin Genet 78:364-372. doi: 10.1111/j.1399-0004.2010.01473.x
